# Longitudinal Circulating Tumor Cell Collection, Culture, and Characterization in Pancreatic Adenocarcinomas

**DOI:** 10.3390/cancers17030355

**Published:** 2025-01-22

**Authors:** Jerry Xiao, Reetu Mukherji, George Sidarous, Shravanthy Suguru, Marcus Noel, Benjamin A. Weinberg, Aiwu He, Seema Agarwal

**Affiliations:** 1 Department of Tumor Biology, Georgetown University, Washington, DC 20057, USA; 2 Department of Internal Medicine, University of California San Francisco, San Francisco, CA 94115, USA; 3 Department of Hematology/Oncology, Medstar Georgetown University Hospital, Washington, DC 20007, USA; 4 Department of Internal Medicine, Medstar Georgetown University Hospital, Washington, DC 20007, USA; 5 Department of Pathology, Georgetown University, Washington, DC 20057, USA

**Keywords:** circulating tumor cells, liquid biopsy, metastasis, transcriptomics, pancreatic adenocarcinomas

## Abstract

Background/Objectives: Pancreatic adenocarcinoma (PDAC) remains one of the most lethal cancers, with limited advancements in treatment efficacy due to high rates of chemoresistance. Circulating tumor cells (CTCs) derived from liquid biopsies offer a non-invasive approach to monitoring tumor evolution and identifying molecular mechanisms of resistance. This study aims to longitudinally collect, culture, and characterize CTCs from PDAC patients to elucidate resistance mechanisms and tumor-specific gene expression profiles. Methods: Blood samples from 10 PDAC patients were collected across different treatment stages, yielding 16 CTC cultures. Differential gene expression, pathway dysregulation, and protein–protein interaction studies were utilized, highlighting patient-specific and disease progression-associated changes. Longitudinal comparisons within five patients provided further insights into dynamic molecular changes associated with therapeutic resistance. Results: CTC cultures exhibited the activation of key pathways implicated in PDAC progression and resistance, including TNFα/NF-kB, hedgehog signaling, and the epithelial-to-mesenchymal transition. Longitudinal samples revealed dynamic changes in signaling pathways, highlighting upregulated mechanisms of chemoresistance, including PI3K/Akt/mTOR and TGF-β pathways. Additionally, protein–protein interaction analysis emphasized the role of the immune system in PDAC progression and therapy response. Patient-specific gene expression patterns therefore suggest potential applications for precision medicine. Conclusions: This proof-of-concept study demonstrates the feasibility of longitudinally capturing and analyzing CTCs from PDAC patients. The findings provide critical insights into molecular drivers of chemoresistance and highlight the potential of CTC profiling to inform personalized therapeutic strategies. Future large-scale studies are warranted to validate these findings and further explore CTC-based approaches in PDAC management.

## Simple Summary

This research focuses on pancreatic adenocarcinomas and takes advantage of novel unbiased culture and characterization models to evaluate circulating tumor cells (CTCs). By collecting and growing CTCs from patients, this study highlights the importance of certain molecular pathways, such as TNF/NF-kB and hedgehog signaling, in cancer progression and treatment failure. These insights could help personalize treatments by identifying specific genetic changes in tumors, making therapies more targeted and effective. This study demonstrates the promise of liquid biopsies for monitoring cancer and shaping future treatment strategies.

## 1. Introduction

Pancreatic cancer remains one of the leading causes of cancer death in the world, with approximately 500,000 new cases diagnosed and 460,000 deaths from pancreatic cancer reported worldwide in 2020 [1]. In the United States, mortality rates have been steadily increasing by 0.3% per year since 2000 [2]. Notably, this steady increase in incidence and mortality rates has occurred despite the incorporation of 5-fluorouracil/leucovorin with irinotecan and oxaliplatin (FOLFIRINOX) and nab-paclitaxel-gemcitabine-based (GemAbra) therapy regimens in metastatic pancreatic adenocarcinoma (PDACs) [3]. These clinical statistics have led researchers to investigate the possible mechanisms of chemoresistance in pancreatic cancer. The results implicate internal signaling pathways, but also external effects such as the tumor microenvironment (TME), in chemo-resistant PDACs, and suggest that there are clinical benefits associated with immune cell infiltration [4,5]. Investigations of patient-derived samples have subsequently identified several key pathways, including AKT/mTOR, WNT/β-Catenin, and TGF-β signaling, among others, as critical pathways involved in chemoresistance to FOLFIRINOX or gemcitabine-based regimens [5,6,7]. On the other hand, numerous targeted therapies, including those aimed at VEGF, PI3K, and IGF-1 receptors, have so far failed to demonstrate improved survival in the clinic despite encouraging pre-clinical data [3]. Increasingly, research on pancreatic cancers and chemoresistance has pointed to the synergism between multiple different pathways, and thus efforts have since necessarily been broadened to evaluate other non-traditional targets for chemoresistance, such as the sirtuin family of NAD+-dependent enzymes [8]. One of the key limitations of identifying broader targets and mechanisms of chemotherapy in pancreatic cancer has been the difficulties in generating appropriate models that are representative of patient samples for study.

In many cancers, liquid biopsies have emerged as an alternative to tissue biopsies. Liquid biopsies are generally a non-invasive and cost-efficient method for the genetic monitoring of a patient’s cancer [9]. One component of liquid biopsies, circulating tumor cells (CTCs), has shown promise for evaluating metastasis-specific phenomena, such as the activation of an epithelial–mesenchymal transition (EMT) [10,11,12]. CTCs are a unique population of cells that have successfully migrated from primary tumors into the bloodstream, enabling the hematologic spread of cancer [10,11,12]. In one study evaluating metastatic breast cancer-derived CTCs from the same patients across two different time points significant changes in expression in multiple clinically relevant genes were identified, including GPRC5D—which was previously associated with tamoxifen resistance—and DNA damage repair genes, that could theoretically play a role in therapeutic responses to alkylating agents [13]. However, a long-standing barrier to the widespread adoption of CTC-based liquid biopsies has been the difficulty in efficiently capturing and growing CTCs for -omics studies [10,14].

Previous studies on PDACs have identified a clinically relevant role for these CTCs as both prognosticators [15,16] and predictors of therapeutic responses [17]. However, these studies are often limited to the enumeration of CTCs, leaving behind a vast array of genomic- and proteomic-level information that are yet to be studied. This practical limitation is due, in part, to the rarity of CTCs, i.e., <5–10 CTCs in a typical 7.5 mL blood draw [18,19]. Therefore, the ability to expand CTCs ex vivo is one strategy to overcome the limitation imposed by the low numbers of CTCs obtained in liquid biopsies. Expanded CTCs can be used to elucidate the genetic mechanisms/signatures of metastasis and gain insight into the evolution of patient-specific tumors. In a previous report, we demonstrated a novel, unbiased method for capturing and propagating metastatic breast cancer-derived CTCs ex vivo, and identified a role for mTORC1 signaling in breast cancer metastasis [14]. We then expanded this technology to evaluate colon-, lung-, and pancreatic-cancer derived CTCs and further emphasized NF-κB and TGF-β signaling in the CTC phenotype [20].

Here, we report the collection, propagation, and evaluation of CTCs derived from patients diagnosed with PDACs. Importantly, we also describe a series of CTCs collected longitudinally from multiple individuals undergoing chemotherapy. Evaluation of the transcriptomics of these cultured CTCs through RNA-sequencing enables an investigation into signaling pathways utilized by patient-derived PDAC cells to escape chemotherapy. Furthermore, protein–protein interaction analysis of highly enriched genes across patient time points highlights the role of chemokine–cytokine signaling and the immune system in PDAC disease progression. Ultimately, this proof-of-concept study demonstrates the potential for patient-derived CTCs to understand the mechanisms of chemoresistance in PDACs. Importantly, the unique gene expression patterns of individual patients suggests that the molecular profiling of CTCs obtained from clinical samples could be leveraged to personalize the treatment of PDACs.

## 2. Materials and Methods

### 2.1. Patient Enrollment

Patients were recruited, consented, and enrolled at the Medstar Georgetown University Hospital Medical Oncology clinics in compliance with the Health Insurance Portability and Accountability Act (HIPAA) and Georgetown University Institutional Review Board (IRB) procedures (approval ID: 2016-0419), and managed through the Survey, Recruitment, and Biospecimen Collection shared resource (SRBSR) of the Lombardi Comprehensive Cancer Center. All patients provided written informed consent for this study. Inclusion criteria for patient enrollment included adults (>18 years old) with histologically confirmed locally advanced or metastatic PDAC. Patients were enrolled into one of three pre-planned cohorts. (1) Patients diagnosed with metastatic PDAC who had progressed on prior line(s) of therapy planned for a single CTC collection. (2) Patients diagnosed with locally advanced PDAC who had ideally received at least 1–2 doses of prior cancer-directed systemic therapy planned for a single CTC collection. (3) Patients newly diagnosed with metastatic PDAC planned for serial CTC collection with the first CTC blood draw ideally prior to initiating front-line treatment for metastatic disease and serial CTC single-sample collections after subsequent lines of therapy, and ideally drawn prior to initiating the next line of therapy if feasible. All CTC collections across cohorts were performed after at least a 1-week interval from the last dose of intravenous systemic therapy. The exclusion criteria included any one or more of the following: non-adenocarcinoma histology, absence of biopsy-proven PDAC diagnosis, and known concurrent secondary malignancy diagnosis. Deidentified genetic reports and clinical annotation regarding patients and patient tumors were provided by the SRBSR.

### 2.2. CTC Capture and Propagation

In all patients enrolled, four tubes of blood (~7–8 mL/tube) were drawn. When applicable and available, blood was drawn from existing IV ports; if blood draws required skin punctures, an initial waste tube was disposed of prior to processing. CTC isolation, capture, and propagation occurred as previously reported [14]. Briefly, patient peripheral blood samples were immediately placed on ice and processed within 90 min of collection from patients. Peripheral blood samples were separated by density using FiColl-Paque (Cytiva Life Sciences, Marlborough, MA, USA, cat. No. 17-440-02) as described previously. All samples were subjected to a 5 min period of red blood cell lysis (Invitrogen, cat. no. 4300-54) prior to plating. Upon completion of the washing and collection steps, cells equivalent to approximately 3–4 mL of patient blood were collected and reserved for RNA extraction (“whole blood matched” or “WBM” samples). RNA (Thermo Fisher, cat. no. AM1912) extraction was performed according to manufacturer’s protocol.

### 2.3. Next-Generation RNA Sequencing Library Preparation and Sequencing

Bulk RNA sequencing library preparation and sequencing was performed with the support of a third-party commercial company, Psomagen (Rockville, MD, USA), as previously described using low-input RNA sequencing [20]. Briefly, libraries were created using TruSeq standard mRNA sample preparation, and paired-end reads with a targeted read length of 151 bp were performed on a NovaSeq 6000 S4 sequencing platform with 50 million reads in each direction. A minimum of 50 ng of RNA was required for processing. Quality assurance of each sample was performed using the following criteria prior to library creation: (1) a minimum of 50 ng of total RNA as assessed by a Qubit 4 Fluorometer; and (2) an RIN value of ≥ 6.0 to move forward with library preparation.

### 2.4. Bulk RNA Sequencing Bioinformatics Analysis

All sequencing files were transferred by Psomagen as raw files. Paired-end read files were first trimmed using Trimmomatic and aligned to the human transcriptome (GRCh38.p13) reference using salmon v0.14. Differential gene expression analysis was performed in R using DESeq2 (version 1.42.1) [21]. Differentially expressed genes were filtered based on the adjusted *p*-value (<0.01), total read counts (>100 reads required total/gene), and adequate samples (>25% of all samples required a count > 10 reads). GSEA and ssGSEA pathway analysis was performed in R using normalized feature counts and the open-source packages fgsea (version 1.28.0). KEGG analysis was also performed in R using the open-source package ClusterProfiler (Version 4.10.1). All data visualization was performed in R using source packages including, but not limited to, ggsci (version 3.1.0), ggplot2 (version 3.5.1), and ComplexHeatmaps (version 2.18.0).

### 2.5. Protein–Protein Interaction Analysis Using STRING-db

Protein–protein interaction analysis was performed via STRING-db (accessed at https://string-db.org (accessed on 1 December 2024)). Log2 fold changes were identified using base R functions and sorted for the highest fold changes. The top 200 genes were then input into the STRING-db database, according to the developer instructions. Gene symbols were matched to the known STRING-db homo sapiens database and subsequently evaluated based on the confidence of their interactions. The minimum required interaction score was set to “high confidence (0.700)”, and clusters were identified via k-means clustering. Relevant biological processes for each cluster were identified based on strength and false discovery rate (FDR < 0.01).

## 3. Results

### 3.1. Establishment of CTC-Derived Pancreatic Cancer Models

Sixteen CTC cultures were established from ten unique patients (clinical information summarized in Table 1) using previously described methods [20]. Importantly, for five patients (Patients 39, 49, 52, 63, and 64), longitudinal samples from different clinical timepoints were captured. Cultures were grown for an average of 40 days (with a range of 30–60 days).

### 3.2. Cultured CTCs Exhibit Activation of EMT, p53, and TNFa Signaling Pathways

**Figure 1:**
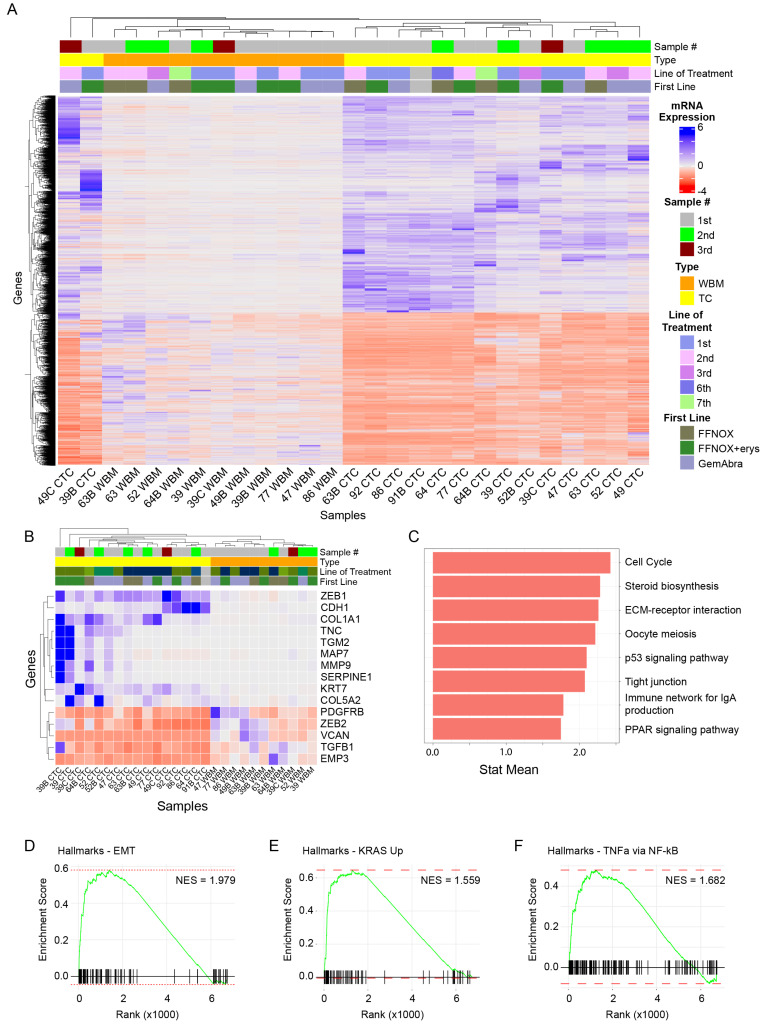
CTC samples were characterized via bulk RNA sequencing, comparing pooled CTC samples against pooled whole blood samples. (**A**) A heatmap of all samples with unbiased clustering demonstrating a clear separation between whole blood samples and processed CTC samples. (**B**) Selected genes associated with epithelial–mesenchymal transitions (EMT) depicted on a heatmap. Among the genes depicted, CTC samples had an overall higher expression of EMT-associated genes, consistent with the known CTC phenotype. (**C**) KEGG pathway analysis using differentially expressed genes between pooled CTCs and whole blood samples. GSEA depicting increased expression of the Hallmarks gene sets associated with (**D**) EMT, (**E**) KRAS signaling, and (**F**) TNFα via NF-κB signaling.

Bulk RNA sequencing and differential gene expression analysis were conducted on all sixteen cultured CTC samples (“TC”) and their corresponding whole blood matched (“WBM”) samples (Figure 1A). Initial analysis comparing WBM to CTC samples identified 2978 downregulated and 4114 upregulated genes in CTCs. MMP7 (Figure S1A, l2fc = 13.93) was the most upregulated gene in CTCs, while DDX1L10 (Figure S1B, l2fc = −7.507) was the most downregulated. Sixteen of the top twenty genes that were upregulated in CTC samples have been previously implicated in metastatic processes across multiple cancer types (Table S1).

CTCs are thought to escape from primary tumors via the activation of an epithelial-to-mesenchymal transition (EMT) [22,23]. Therefore, we carried out a sub-analysis that focused on genes known to be involved in EMT. Cultured CTCs demonstrated an overall upregulation of EMT-associated genes, such as ZEB1, CDH1, COL1A1, and SERPINE1 (Figure 1B). Additionally, there has been a growing interest in identifying clinically relevant biomarkers to risk-stratify patients with PDACs [24,25,26]. In these samples, biomarkers that were associated with poorer overall survival, such as CEP55, KIF4A, CDC45, ASPM, and E2F7, were overexpressed in CTCs relative to WBM samples (Figure S2).

Next, we sought to determine if there were any broad regulatory changes in signaling pathways between CTCs and WBM samples. The Kyoto Encyclopedia of Genes and Genomes (KEGG) contains a collection of gene catalogs related to various important biological functions and signaling pathways [27]. Notably, among the most upregulated gene sets in CTCs were the p53 signaling (hsa04115, *p*-value = 0.0195) and PPAR signaling (hsa03320, *p*-value = 0.0435) pathways (Figure 1C), in addition to JAK-STAT signaling (hsa04630). KEGG findings were supported by gene set enrichment analysis (GSEA) [28]. When comparing CTCs against WBM samples, 11 gene sets were significantly (*p*-adjusted < 0.01) upregulated, including the gene sets for EMT (Figure 1D, NES = 1.979), KRAS signaling (Figure 1E, NES = 1.559), mTORC1 signaling (NES = 1.648), hypoxia (NES = 1.735), and TNFa signaling via NF-kB (Figure 1F, NES = 1.682).

### 3.3. Longitudinal Patient Series Elucidate Potential Resistance Mechanisms

**Figure 2:**
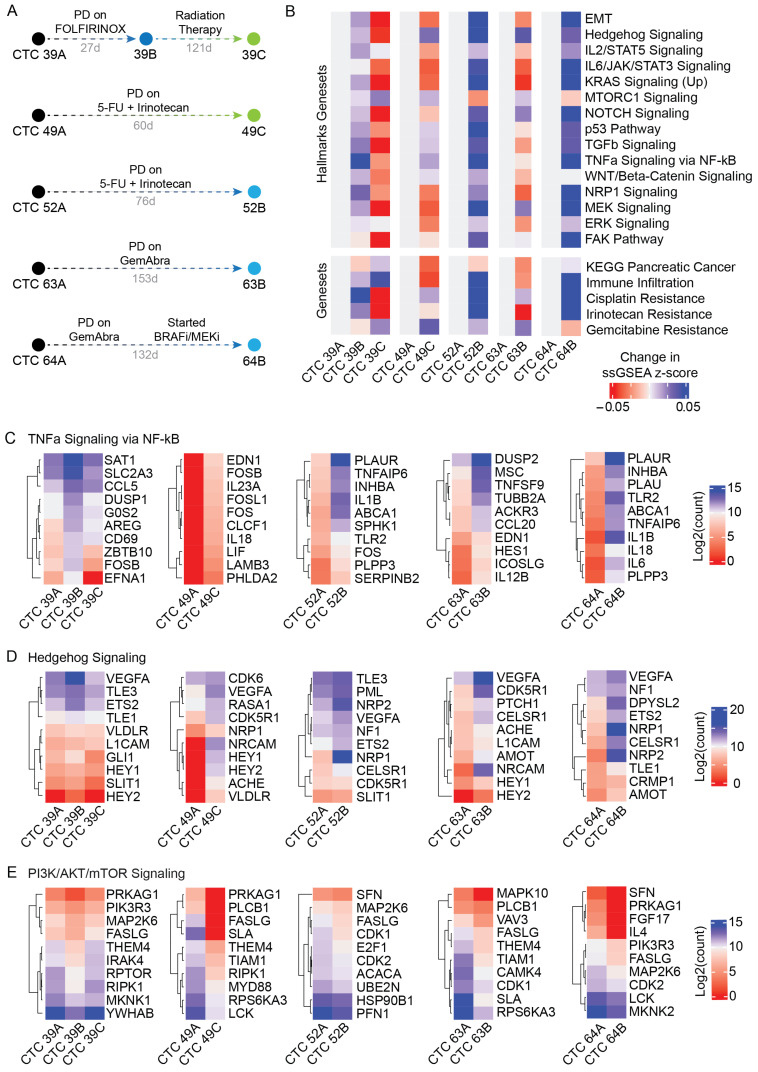
Longitudinal collection of samples from five individuals. (**A**) General timeline of collection of longitudinal samples from the five individuals. More detailed timelines can be found in the supplemental figures. (**B**) A curated list of 15 signaling pathways and 5 broader gene-sets was evaluated using ssGSEA across all longitudinally collected samples, demonstrating various changes across signaling pathways and across therapies. Heatmaps depicting the log2-fold count of the 10 most differentially expressed genes in (**C**) TNFα via NF-κB signaling, (**D**) hedgehog signaling, and (**E**) PI3K/AKT/mTOR signaling within each pairwise comparison.

We also collected CTCs from different time points for five patients (Patients 39, 49, 52, 63, and 64), allowing pairwise comparisons (Figure 2A). From Patient 39, samples were collected: (i) one month after initiating first-line treatment with FOLFIRINOX + investigational agent eryaspase (suspension of erythrocytes encapsulating L-asparaginase) (“CTC 39A”); (ii) two months into treatment amidst a rising CA 19-9 and stable CT scans (“CTC 39B”); and (iii) two days after the completion of five doses of radiation therapy (“CTC 39C”) (Figure S3A). Patient 49, with metastatic *KRAS* G12R, *TP53* R306, and *ARID1A* E2054*-positive PDAC with hepatic, lung, and renal metastases, had three samples collected, but only two usable samples spanning a 60-day period, during which the patient progressed while on combinatorial 5-FU and irinotecan therapy (Figure S3B). Patient 52, diagnosed with locally advanced PDAC, provided two samples over 76 days while on similar 5-FU- and irinotecan-based therapy (Figure S3C). Patients 63 and 64, both with metastatic PDACs, had samples reflecting disease progression while on GemAbra therapies (Figure S3D–E). Notably, Patient 63 had a *KRAS* G12V, *SMAD4*, *GNAS*, and *KMT2C* mutant-positive PDAC with liver metastases while Patient 64′s cancer was *BRAF* V600E-positive, and they had also undergone treatment with experimental BRAF and MEK inhibitors (Figure S3E).

We hypothesized that dysregulation across samples during disease progression could highlight pathways crucial for PDAC progression or therapeutic resistance. Because CTC samples are rare and difficult to collect, single-sample GSEA (ssGSEA) was used to calculate enrichment scores for a curated list of 15 signaling gene sets and 5 broader gene sets encompassing pancreatic cancer and various chemoresistances (Figure 2B) [29]. Globally, of the six pairwise comparisons described above, all but the transition from CTC 39B to 39C represent disease progression while on treatment. Using ssGSEA, three signaling pathways showed consistent dysregulation: TNFα signaling via NF-κB (Figure 2C) and hedgehog signaling (Figure 2D), which were consistently increased; and PI3K/Akt/mTOR signaling (Figure 2E), which was consistently decreased.

**Figure 3:**
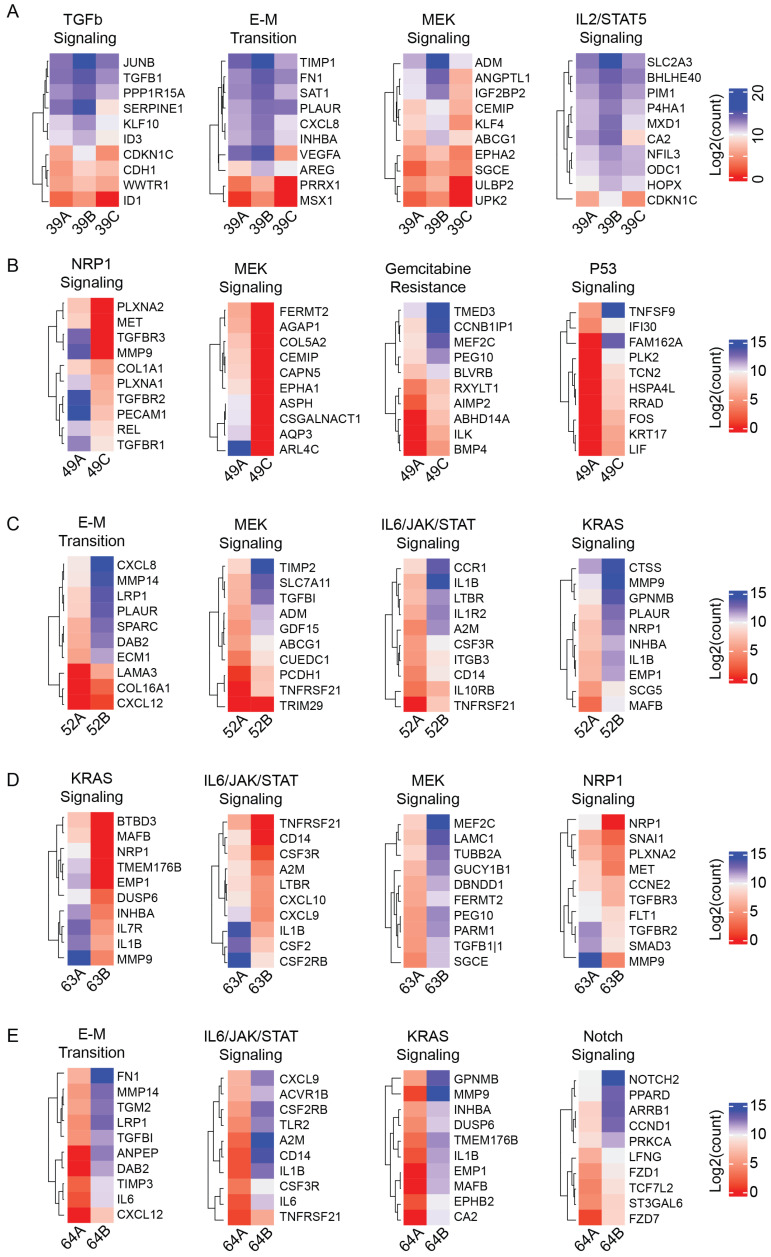
ssGSEA evaluation of individual pairwise comparisons reveal intra-patient specific dysregulation of various signaling pathways. The heatmaps depicted represent the top 10 most dysregulated genes within each specified pathway. Pathways were selected to be the among the four most dysregulated pathways (not including TNFα via NF-κB, Hedgehog, or PI3K/AKT/mTOR signaling) within (**A**) patient 39, (**B**) patient 49, (**C**) patient 52, (**D**) patient 63, and (**E**) patient 64.

Cancer heterogeneity is often implicated in chemoresistance [5]. Our individual pairwise comparisons highlight this variability. For instance, the transition from samples CTC 39A to 39B, which represents disease progression while on FOLFIRINOX + eryaspase treatment, demonstrated increases in not just the aforementioned TNFα signaling, but also TGFβ (Δ = 0.0274), EMT (Δ = 0.0196), and MEK signaling (Δ = 0.0184), all three of which have been previously implicated in FOLFIRINOX resistance (Figure 3A) [5,30]. On the other hand, while the transitions from CTC 49A to 49C and CTC 52A to 52B both encompassed disease progression in the setting of combinatorial 5-FU- and Irinotecan-based therapies, these comparisons revealed opposing changes in MEK signaling (Δ = −0.0399 in patient 49; Δ = 0.0515 in patient 52) (Figure 3B,C). Meanwhile, in comparing CTC 63A to 63B, which spanned the progression of disease while on GemAbra-based therapy, ssGSEA revealed a broad downregulation of many signaling pathways, including KRAS signaling (Δ = −0.0469) and IL6/JAK/STAT3 signaling (Δ = −0.0390), with the converse enrichment of MEK (Δ = 0.0279) signaling (Figure 3D). Finally, evaluation of the transition between CTC 64A and 64B, which was collected following treatment with experimental BRAF and MEKi inhibitors, revealed a persistent elevation in the same IL6/JAK/STAT3 (Δ = 0.0972), and KRAS (Δ = 0.0939) signaling pathways (Figure 3E). While no clear trend emerges from these individual comparisons, what becomes clear is that there remains a plethora of canonical pathways that enable chemoresistance in PDAC.

Finally, we sought to characterize the expected protein–protein interactions of highly enriched genes within each patient to identify the cornerstones of the transcriptomic network involved in metastasis and chemoresistance. STRING is a protein network meta-resource designed to predict protein–protein functional linkage data and enable the visualization and identification of functionally enriched processes/pathways [31]. Within each pairwise comparison, we evaluated the top 200 genes with increased expression between transition points and evaluated for clustering based on known interactions. Clusters of genes involved in chemokine-mediated signaling and/or immune response were routinely identified in comparisons of differentially expressed genes between CTC 39A to 39B (Figure S4A, “cluster A”, FDR = 1.53 × 10^−6^), CTC 49A to 49C (Figure S4B, “cluster A”, FDR = 1.4 × 10^−2^), CTC 52A to 52B (FDR = 7.67 × 10^−21^), and CTC 63A to 63B (Figure S4D, “cluster A”, FDR = 1.21 × 10^−12^). Other identified clusters involved natural killer cell-mediated cytotoxicity in CTC 49A to 49C (Figure S4B, “cluster B”, FDR = 4.8 × 10^−4^), G2/M1 cell-cycle transition in CTC 52A to 52B (Figure S4C, “cluster A”, FDR = 4.9 × 10^−3^), and IL-1-related activity in CTC 63A to 63B (Figure S4D, “cluster B”).

## 4. Discussion

Here, we report the growth and evaluation of gene expression by RNA sequencing of 16 CTC samples collected from 10 unique patients. Attempts to capture and grow CTCs from pancreatic cancers have often been met with low efficiency [32,33,34]. In a previous report and in this study, we have demonstrated success in establishing long-term CTC cultures from patients with metastatic or locally advanced PDACs [14,20]. Global evaluation of differentially expressed genes across the pool of CTC cultures from this study compared to patient-matched whole blood samples identified enrichment in EMT markers in cultured CTCs, consistent with the expected phenotype of CTCs [20,22]. For instance, the most upregulated gene across all samples was MMP-7, a member of the matrix metalloproteinase family that is often cited to be involved in cell proliferation and tumorigenesis. In pancreatic cancers, the knock-out of MMP-7 in genetically engineered murine models resulted in decreased lymph node metastasis formation [35]. In addition, PDAC cell lines treated with oxaliplatin have been correlated with increased MMP-7 expression, promoting survival through the MAPK signaling pathway [36].

Furthermore, in this population of PDAC-derived CTCs, we identified the enrichment of pathways involved in the tumorigenesis of PDACs, including p53 (Figure 1C) and KRAS (Figure 1E) signaling [37]. Furthermore, we also uncovered the enrichment of PPAR signaling, which has increasingly been recognized for its tumorigenic potential (Figure 1C) [38]. Notably, PPARα-based therapies such as clofibrate and fenofibrate have already been investigated for their anti-cancer effects in pancreatic cancer, with promising pre-clinical evidence in both cell lines and animal models [39]. Future studies of these compounds in patient-derived samples, such as the CTCs described here, may offer a more biologically relevant model to evaluate therapies targeting PPARα.

Another potential benefit of liquid biopsies is the ability to routinely monitor patient disease progression with relative ease [10]. Here, we captured and profiled multiple CTC samples from five patients undergoing varying chemotherapy regimens (Figure 2A and Figure S3). Across all five comparisons spanning disease progression while on therapy, we uncovered increased expression of TNFα/NF-κB signaling and hedgehog signaling, as well as an opposing downregulation of PI3K/Akt/mTOR signaling (Figure 2C–E). These findings are largely consistent with the previously reported changes associated with chemoresistance and disease progression in PDACs [5]. The activation or upregulation of NF-κB via multiple routes, including but not limited to the TME, tumor-associated macrophages, or oncogenic mutations in *KRAS*, has been shown to favor cancer tumorigenesis, angiogenesis, and metastasis [40]. As previously mentioned, NF-κB signaling has also been strongly implicated in acquired resistance towards gemcitabine via a multitude of downstream pathways. While preclinical studies evaluating the effects of NF-κB inhibitors in cancers have demonstrated promising results, many of these have not translated clinically due to the broad range of normal cellular homeostatic activities related to NF-κB [40]. Similarly, another signaling pathway commonly activated by *KRAS* mutations and present across multiple cancer types is the PI3K/AKT/mTOR signaling pathway via *PI3K* and *Ras* activation by phosphorylation [41]. This activation has led to multiple studies evaluating targeted therapies against the PI3K/AKT/mTOR pathway, with mixed results depending on the cancer type [41].

Of the three pathways that we report here, hedgehog signaling is perhaps the least understood within the context of pancreatic cancer [42]. Overexpression of the sonic hedgehog (*SHH*), one of three ligands that regulate the hedgehog signaling pathway, has been observed in both pre-invasive and invasive epithelium in human pancreatic cancer samples [43]. In early studies, it was postulated that aberrant hedgehog signaling in pancreatic cancer was the result of constitutive NF-κB signaling driven by oncogenic *KRAS* expression [43]. Unfortunately, follow-up studies involving the knock-out of hedgehog signaling in the pancreas resulted in little to no effect on development of PDACs [44]. Currently, attention regarding hedgehog signaling has turned to its activation by cancer-associated fibroblasts (CAFs) and the associated modulation of the TME [45]. Regardless of the underlying mechanism, the presence of consistently upregulated hedgehog signaling in the CTC samples studied here continues to suggest an important role for hedgehog signaling in pancreatic cancer, cancer metastases, and disease progression.

While evaluating trends in signaling pathways across patients can help inform broader CTC-specific phenomena, there remains a plethora of data that can be gathered from inter-patient comparisons. In patient 39, a comparison of samples collected during disease progression while on combinatorial FOLFIRINOX + eryaspase therapy also revealed increased expression of TGF-β and MEK signaling, both of which have strong associations with chemoresistance to FOLFIRINOX-based therapies in pancreatic cancers through WNT/β-catenin signaling or RAF/MEK/ERK signaling cascades, respectively (Figure 3A) [6].

Of note, patient 52 was the only locally advanced PDAC evaluated in this study, as well as the only patient who had a prior history of a secondary malignancy. Samples collected from patient 52 spanned a 76-day period, during which the patient experienced disease progression while on combined 5-FU and irinotecan therapy (Figure 2A). 5-FU acts in an anti-cancer mechanism through the disruption of multiple cellular functions, including apoptosis, EMT activation, glucose metabolism, and cellular division [30]. Additionally, it has been proposed that the TME also contributes to 5-FU chemoresistance through the activation of the PI3K/Akt/mTOR and MEK/ERK pathways via IGF-1R secretion [30]. These pre-clinical studies led to the development of multiple-phase I/II clinical trials investigating anti-IGF-1R therapies in PDACs [46]. In our study, these phenomena are also observed, with increased expression of PI3K/Akt/mTOR, and MEK signaling pathways in the 5-FU-treated CTC 52B sample relative to the baseline (Figure 3C).

One of the most used and well-studied regimens in PDACs involves gemcitabine-based therapies [3]. Two samples from patient 63 were collected in the setting of GemAbra therapy amidst a rising CA 19-9 (Figure 2A). Patient 63 was one of the few samples in this study to have demonstrated a globally reduced expression of the KEGG pathway associated with pancreatic cancer, which may have signaled a mixed response to treatment (Table S2). The pathway dysregulation observed in patient 63 also revealed evidence consistent with gemcitabine resistance. Proposed mechanisms of Gemcitabine chemoresistance have included the upregulation of NOTCH2, the constitutive activation of NF-κB leading to the activation of Notch and Hedgehog signaling, and the overexpression of oncomucins [47]. Similarly, genetic analyses of our CTC samples indicated increases in both Notch and Hedgehog signaling, with only a minimal increase in NF-κB signaling (Figure 3D, Table S2). On the other hand, while prior studies have implicated activation of the IL-6/IL-6R/STAT3 axis in gemcitabine-based chemoresistance, we found that IL-6/JAK/STAT3 signaling was decreased following exposure to gemcitabine (Figure 3D).

Compared to the other patients evaluated, patient 64 represented a unique genetic background involving the presence of a *BRAF* V600E mutation. *BRAF* mutations are relatively rare in PDACs, with an incidence rate of approximately 3% [48]. Prior to enrollment in this study, the patient had undergone targeted BRAF therapy with dabrafenib/trametinib and was on a second rechallenge treatment with an ERK inhibitor when CTC 64B was collected (Figure S4E). Mechanistically, the *BRAF* V600E mutation enhances kinase activity, resulting in the downstream activation of the MAPK pathway and the suppression of cell apoptosis and increased tumorigenesis [49]. This mechanism is supported by our study, where we observed a persistent increase in KRAS signaling, which was observed despite 5 months of therapy (Figure 3E). Both the MEK and ERK signaling pathways were also enriched despite ongoing ERK1/2 inhibitor therapy (Table S2). ERK activation in response to extracellular stimuli or RAS-activating mutations is commonly associated with a more aggressive phenotype in tumors as well as chemo-resistance mechanisms [50]. We suggest that the observed increases in MEK and ERK signaling pathways despite ERK inhibitor therapy could represent incomplete ERK inhibition or otherwise overwhelming chemo-resistance in this patient’s PDAC.

Another emerging phenomenon that was observed in this study involves recruitment of the immune system by PDACs to escape chemotherapy via the attraction of tumor-associated macrophages and the promotion of regulatory T-cells via the surrounding TME [51]. In this study, we used protein–protein interaction analysis via STRING to investigate the function of the genes that were the most upregulated in post-treatment samples. Cluster analysis carried out on each individual patients studied invariably revealed clusters associated with immune infiltration in pancreatic cancer as well as chemokine- or cytokine-related signaling (Figure S4). Thus, the prevalence of immune-related activity in all patients, irrespective of treatment type or individual patient genetics, continue to suggest a broad and overarching interaction between the immune system and PDACs that remains to be explored.

This study has limitations. While many of the findings in this study are consistent with known results obtained via tissue biopsies, there remains outliers (e.g., CTC 52, a locally advanced PDAC with a prior history of resected colon cancer) which cannot be confirmed at this time due to the small cohort size studied. Additionally, given the nature and rarity of CTCs, we were limited in pathway analysis to single-sample gene set enrichment analysis (ssGSEA) [28]. Future studies should seek to expand upon this study, in both the number of samples per patient as well as the number of patients, to further improve statistical power. However, despite these limitations, this proof-of-concept study has successfully demonstrated the potential of longitudinal monitoring of patient tumors through CTCs.

## 5. Conclusions

Overall, this study involving the capture, propagation, and characterization of 16 CTC samples from 10 patients diagnosed with PDAC demonstrates both (a) the capabilities of an efficient system for studying CTCs and (b) the genetic and molecular information that can be gleaned from long-term monitoring of CTCs in patients. For the five patients from which multiple CTC samples were collected, we identified patterns in signaling pathway dysregulation that were consistent with known roles in chemoresistance or disease progression, including enrichment of TNFα/NF-κB signaling and hedgehog signaling, as well as opposing downregulation of PI3K/Akt/mTOR signaling in PDAC metastasis. Furthermore, protein–protein interaction analysis of these samples reinforced the role of the immune system in both disease progression and chemoresistance. Future studies scaling the method utilized here could further reveal the genetic drivers of PDAC metastasis, identify chemoresistance mechanisms, and highlight potential therapeutic targets to limit the spread of cancer. Therefore, this study serves as a suitable template from which future larger-scale interrogation of CTCs obtained from PDAC patients could be conducted.

## Figures and Tables

**Table 1:** Table of characteristics for enrolled patients.

ID #	Series	Age/Gender	Clinical Stage	Metastasis Location	Prior RT ^1^/IR ^2^?
**39**	A	74 yo M	Metastatic	Abdominal Pelvic LN Lung	N
	B				N
	C				Y
**47**		67 yo F	Metastatic	Abdominal Pelvic LN Liver Spleen Adrenal	N
**49**	A	70 yo M	Metastatic	Liver Peritoneum	N
	C				N
**52**	A	73 yo F	Locally advanced	Colon	Y
	B				Y
**63**	A	68 yo M	Metastatic	Abdominal Pelvic LN Liver Lung	Y
	B				Y
**64**	A	54 yo F	Metastatic	Abdominal Pelvic LN Thoracic LN Liver Peritoneum Skin Spine Breast	N
	B				N
**77**		73 yo F	Metastatic	Peritoneum	N
**86**		68 yo M	Locally advanced		N
**91**	B	59 yo F	Metastatic	Abdominal Pelvic LN Liver Peritoneum Lung	N
**92**		60 yo F	Locally advanced		N

^1^ RT = radiation therapy. ^2^ IR = infrared therapy.

## Data Availability

The datasets used and/or analyzed during the current study are available from the corresponding author on reasonable request.

## Supplementary Materials

The following information can be downloaded at: https://www.mdpi.com/article/10.3390/cancers17030355/s1, Figure S1: Clustergrams depicting the 20 most (A) upregulated and (B) downregulated genes in cultured circulating tumor cell (CTC) samples relative to whole blood matched (WBM) samples. Figure S2: CTC samples routinely exhibited increased expression in previously described pancreatic cancer biomarkers, consistent with the understanding that CTC resemble the most aggressive forms of their corresponding cancers. Figure S3: Detailed timelines capturing treatments, radiation therapy, scan results, and CA 19-9 levels for patients (A) 39, (B) 49, (C) 52, (D) 63, and (E) 64. Figure S4: Protein-protein analysis was conducted using string-DB to predict transcriptomic networks involved in metastasis and chemoresistance. The top 2-4 clusters with > 3 genes are depicted from the comparisons between (A) 39A to 39B, (B) 49A to 49B, (C) 52A to 52B, and (D) 63A to 63. Table S1: Literature study evaluating the top 20 most upregulated genes in CTC samples demonstrates broad implication in cancer metastasis and oncogenesis. Table S2: ssGSEA scores comparing the six pairwise comparison.

## Abbreviations

The following abbreviations are used in this manuscript:

CTC	Circulating tumor cells
PDAC	Pancreatic ductal adenocarcinoma
FOLFIRINOX	5-Flurouracil/Leucovorin with irinotecan and oxaliplatin
EMT	Epithelial–mesenchymal transition
HIPAA	Health Insurance Portability and Accountability Act
IRB	Institutional Review Board
SRBSR	Survey, Recruitment, and Biospecimen Collection Shared Resource
KEGG	Kyoto Encyclopedia of Genes and Genomes
GSEA	Gene set enrichment analysis
GemAbra	Gemcitabine and Abraxane-based therapy
