# Supplementary material for: Longitudinal Circulating Tumor Cell Collection, Culture, and Characterization in Pancreatic Adenocarcinomas
Source: Cancers (Basel). 2025 Jan 22;17(3):355. doi: 10.3390/cancers17030355 (PMC11815863; doi:10.3390/cancers17030355)
Supplement: Supplementary file 1 [file cancers-17-00355-s001.zip › cancers-3405927-supplementary.pdf]

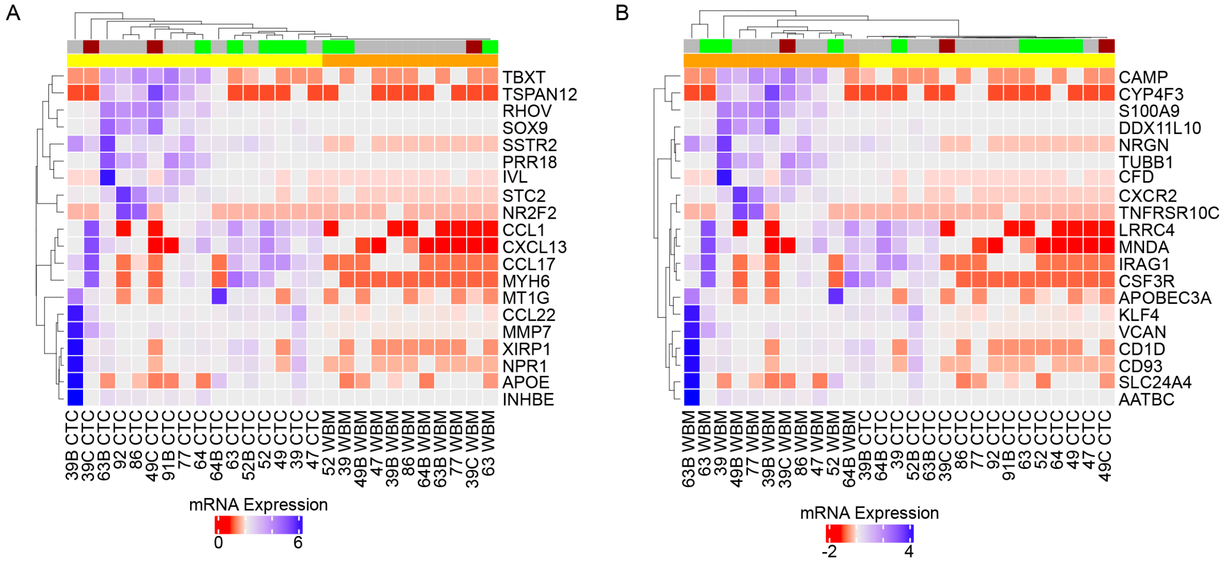

Figure S1 – Clustergrams depicting the 20 most (A) upregulated and (B) downregulated genes in cultured circulating tumor cell (CTC) samples relative to whole blood matched (WBM) samples.

Table S1 – Literature study evaluating the top 20 most upregulated genes in CTC samples demonstrates broad implication in cancer metastasis and oncogenesis.

Gene	Previous Studies
TBXT	- Targeted inhibition of TBXT with miR-219-5p resulted in cell migration and invasion of breast cancer cells. [1]
TSPAN12	- Higher expression of TSPAN12 clinically correlated with colorectal cancer growth and metastasis. [2]
RHOV	- RHOV activation of the JNK/c-Jun signaling pathway in lung cancers promotes lung adenocarcinoma growth and metastasis. [3]<br>- Gain-of-function studies using a CRISPR screen demonstrated a pro-metastatic role for RhoV in triple-negative breast cancers. [4]
SOX9	- SOX9, a member of the HMG-box transcription factors, has been consistently shown to be an important regulator of breast cancer growth and metastasis *in vivo*. [5]
SSTR2	- High expression of SSTR2, a member of the somatostatin receptors, has been trialed as a therapeutic and diagnostic biomarker for neuroendocrine tumors. [6]
PRR18	*Not previously mentioned in metastatic processes*.
IVL	- IVL belongs to a set of "hub genes," including KRT5 and DSP, that were identified by bioinformatics analysis as genes important to the metastatic process for melanoma. [7]
STC2	- STC2 is a broadly expressed protein across a spectrum of tumor tissues—most notably, overexpression of STC2 correlated with tumor growth and invasion in colorectal cancers. [8]
NR2F2	- NR2F2 acts as an epigenetic modulator of EMT- and NCC-associated target genes in metastatic melanoma. [9]
CCL1	- Recombinant CCL1 promotes migration of CCR8-positive malignant melanoma cells resulting in increased lymph node metastasis in mouse models. [10]
CXCL13	- CXCL13 plays a role in the tumor microenvironment. Expression of CXCL13 was associated with increased tumor infiltration by CXCR5-expressing CD8+ T cells. [11]
CCL17	- CCL17 expression in lung adenocarcinomas is positively correlated with the presence of tumor-infiltrating lymphocytes, immunostimulators, and major histocompability complexes. [12]
MYH6	*Not previously studied in pancreatic cancer metastasis*.
MT1G	- MT1G expression has previously been linked to suppressed carcinogenesis and inhibition of metastasis in pancreatic cancer cells. [13]
CCL22	- CCL22 is most commonly expressed on the surface of tumor-associated macrophages found in advanced melanoma and esophageal squamous cell carcinoma. [14]
MMP7	- High-levels of MMP7 expression in circulation is associated with poor prognosis. MMP7 is thought to increase pancreatic cancer cell migration and proliferation. [15]
XIRP1	*Not previously studied in pancreatic cancer metastasis.*
NPR1	- NRP1 expression predicts poor prognosis through activation of the EMT and PI3K/Akt signaling pathways in gastric cancers. [16]
APOE	- APOE promotes tumor metastasis in colorectal cancers through the JUN signaling pathway. [17]
INHBE	*Not previously studied in pancreatic cancer metastasis*.

Table S2 - ssGSEA scores comparing the six pairwise comparison

	39A -> B	39B -> C	49 A -> C	52 A -> B	63 A -> B	64 A -> B
EMT	0.0196	-0.103	-0.0347	0.0880	-0.0287	0.125
Hedgehog Signaling	0.0130	-0.0598	0.0313	0.0506	0.0419	0.0351
IL2/STAT5 Signaling	0.0193	-0.0178	-0.0222	0.0128	-0.0139	0.0226
IL6/JAK/STAT3 Signaling	0.000589	-0.0378	-0.0398	0.0463	-0.0390	0.0972
KRAS Signaling	0.0175	-0.0676	-0.0371	0.0546	-0.0469	0.0939
Hallmark_Mitotic Spindle	0.00178	-0.0106	-0.00950	-0.0312	-0.0234	0.00376
mTORC1 Signaling	0.00506	0.0217	0.0138	-0.0263	0.00989	-0.0101
NOTCH Signaling	0.0112	-0.0887	-0.0124	0.0401	0.0265	0.0791
P53 Pathway	0.0188	-0.0458	0.00782	0.0505	-0.00427	0.0390
PI3K/AKT/mTOR Signaling	-0.0129	0.0246	-0.0222	-0.0133	-0.00791	-0.000879
TGFβ Signaling	0.0274	-0.0855	0.00884	0.0414	-0.0228	0.0392
TNFα via NF-κB Signaling	0.0555	-0.0803	0.0179	0.0557	-0.00324	0.0827
WNT/β-Catenin Signaling	0.0141	-0.0459	0.00468	0.0166	-0.0153	0.000214
ERK Signaling	0.00357	-0.00279	-0.0307	0.00962	-0.0252	0.0122
FAK Pathway	-0.00304	-0.0738	-0.00439	0.0391	0.001545	0.0679
NRP1 Signaling	0.0338	-0.0589	-0.0313	0.0254	-0.0377	0.0514
MEK Signaling	0.0184	-0.0893	-0.0399	0.0515	0.0279	0.0767
Immune Infiltration	0.0140	0.0332	-0.0435	0.0570	-0.0254	0.0866
KEGG Pancreatic Cancer	-0.00954	0.0199	-0.0381	-0.00893	-0.0282	0.00266
Cisplatin Resistance	0.0634	-0.139	0.0173	0.113	0.0141	0.124
Irinotecan Resistance	0.0261	-0.145	-0.00451	0.0627	-0.0861	0.0678
Gemcitabine Resistance	-0.00376	0.0282	0.0392	0.0148	0.0287	-0.0164

A

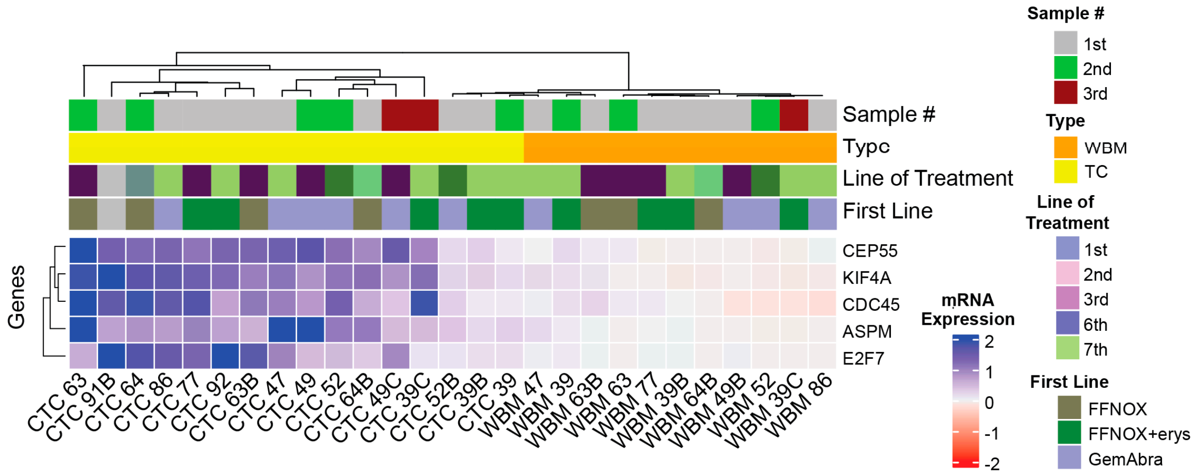

Figure S2 – CTC samples routinely exhibited increased expression in previously described pancreatic cancer biomarkers, consistent with the understanding that CTC resemble the most aggressive forms of their corresponding cancers.

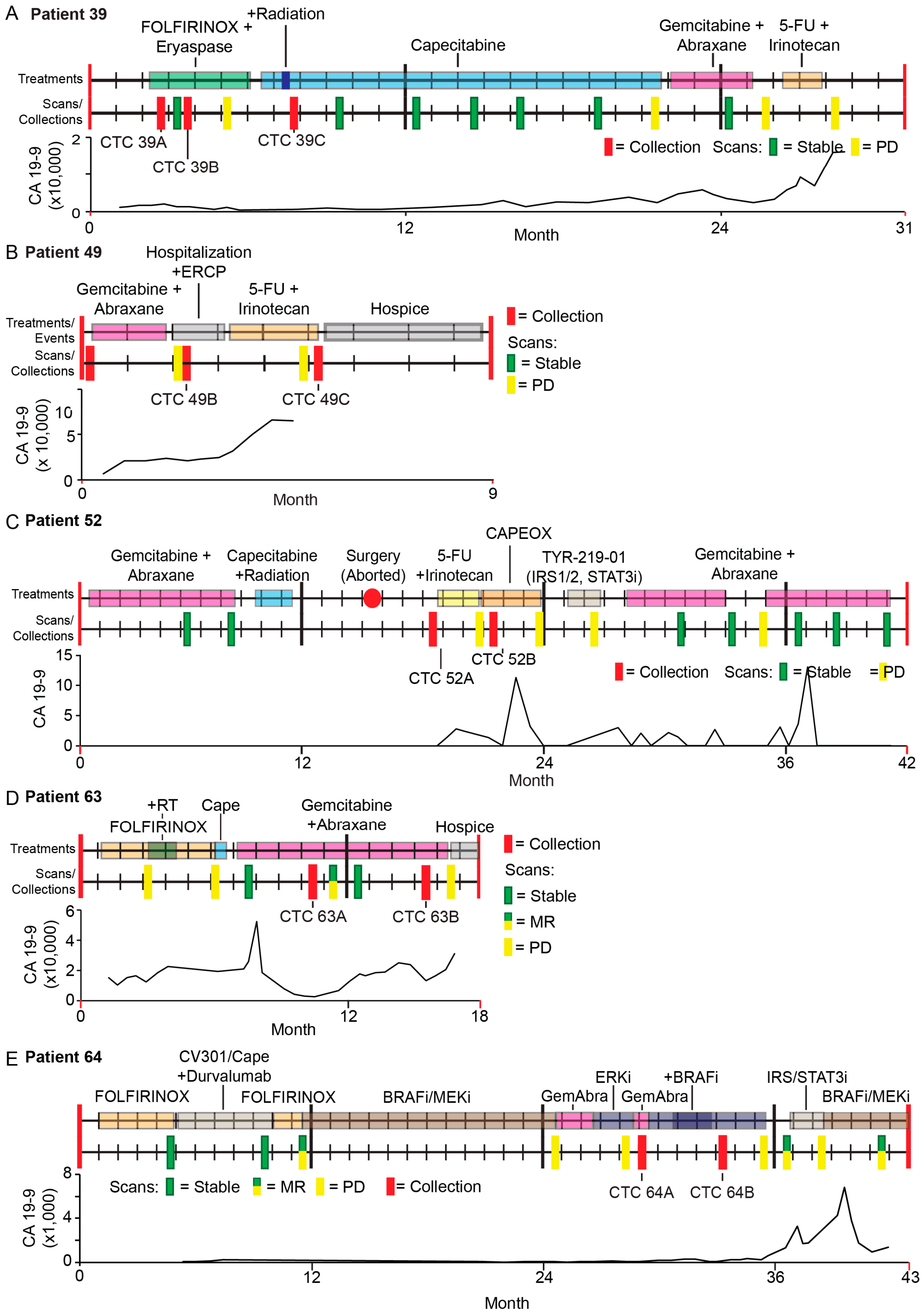

Figure S3 – Detailed timelines capturing treatments, radiation therapy, scan results, and CA 19-9 levels for patients (A) 39, (B) 49, (C) 52, (D) 63, and (E) 64.

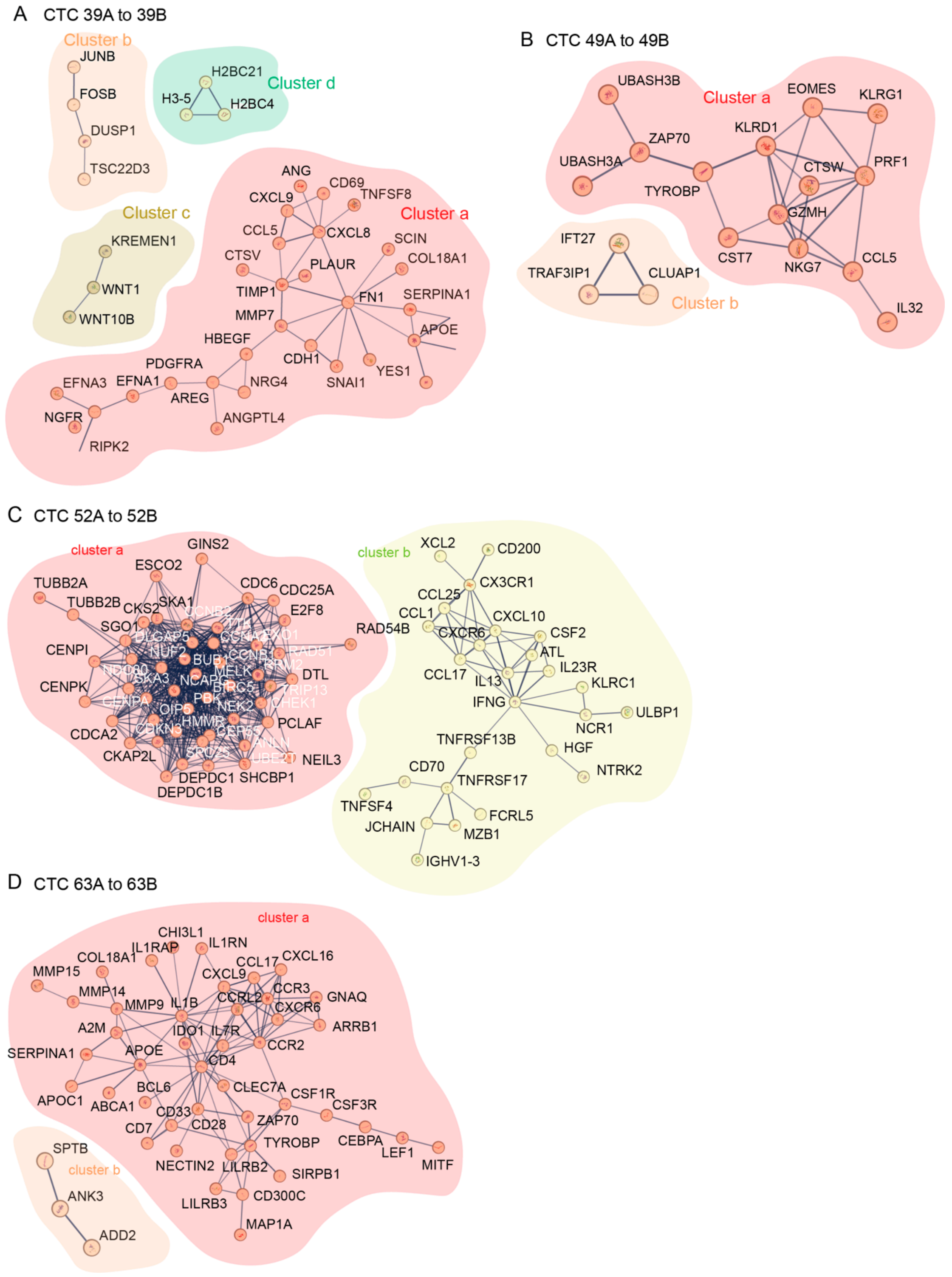

Figure S4 – Protein-protein analysis was conducted using string-DB to predict transcriptomic networks involved in metastasis and chemoresistance. The top 2-4 clusters with > 3 genes are depicted from the comparisons between (A) 39A to 39B, (B) 49A to 49B, (C) 52A to 52B, and (D) 63A to 6
